# Supplementary material for: Host-cell Interactions of Engineered T cell Micropharmacies
Source: bioRxiv. 2023 May 1:2023.04.05.535717. Preprint. [Version 2] doi: 10.1101/2023.04.05.535717 (PMC10187158; doi:10.1101/2023.04.05.535717)
Supplement: Supplement 1 [file NIHPP2023.04.05.535717v2-supplement-1.pdf]

bioRxiv preprint doi: https://doi.org/10.1101/2023.04.05.535717; this version posted May 1, 2023. The copyright holder for this preprint (which was not certified by peer review) is the author/funder, who has granted bioRxiv a license to display the preprint in perpetuity. It is made available under aCC-BY 4.0 International license.

Supplemental for: Host-cell Interactions of Engineered T cell Micropharmacies

Host-cell Interactions of Engineered T cell Micropharmacies

Christopher M. Bourne[1], Patrick Wallisch[2], Megan Dacek[2], Thomas Gardner[3], Kristen Vogt[4], Broderick C. Corless[2], Mamadou A. Bah[1], Jesus Romero Pichardo[5], Angel Charles[3], Keifer G. Kurtz[2], Derek S. Tan[2,4,5,6], David A. Scheinberg[2,3,4,5, 7]

1.) Immunology and Microbial Pathogenesis Program, Weill Cornell Graduate School of Biomedical Sciences, Memorial Sloan Kettering Cancer Center, New York, NY, USA 10065.

2.) Pharmacology Program, Weill Cornell Graduate School of Biomedical Sciences, Memorial Sloan Kettering Cancer Center, New York, NY, USA, 10021.

3.) Molecular Pharmacology Program, Memorial Sloan Kettering Cancer Center, New York, NY, USA 10065.

4.) Tri-Institutional PhD Program in Chemical Biology, Memorial Sloan Kettering Cancer Center, New York, NY, USA, 10065.

5.) Louis V. Gerstner Jr. Graduate School of Biomedical Sciences, Memorial Sloan Kettering Cancer Center, New York, NY 10065, USA

6.) Chemical Biology Program, Sloan Kettering Institute, Memorial Sloan Kettering Cancer Center, New York, NY, USA, 10065.

7.) Correspondence address: scheinbd@mskcc.org

bioRxiv preprint doi: https://doi.org/10.1101/2023.04.05.535717; this version posted May 1, 2023. The copyright holder for this preprint (which was not certified by peer review) is the author/funder, who has granted bioRxiv a license to display the preprint in perpetuity. It is made available under aCC-BY 4.0 International license.

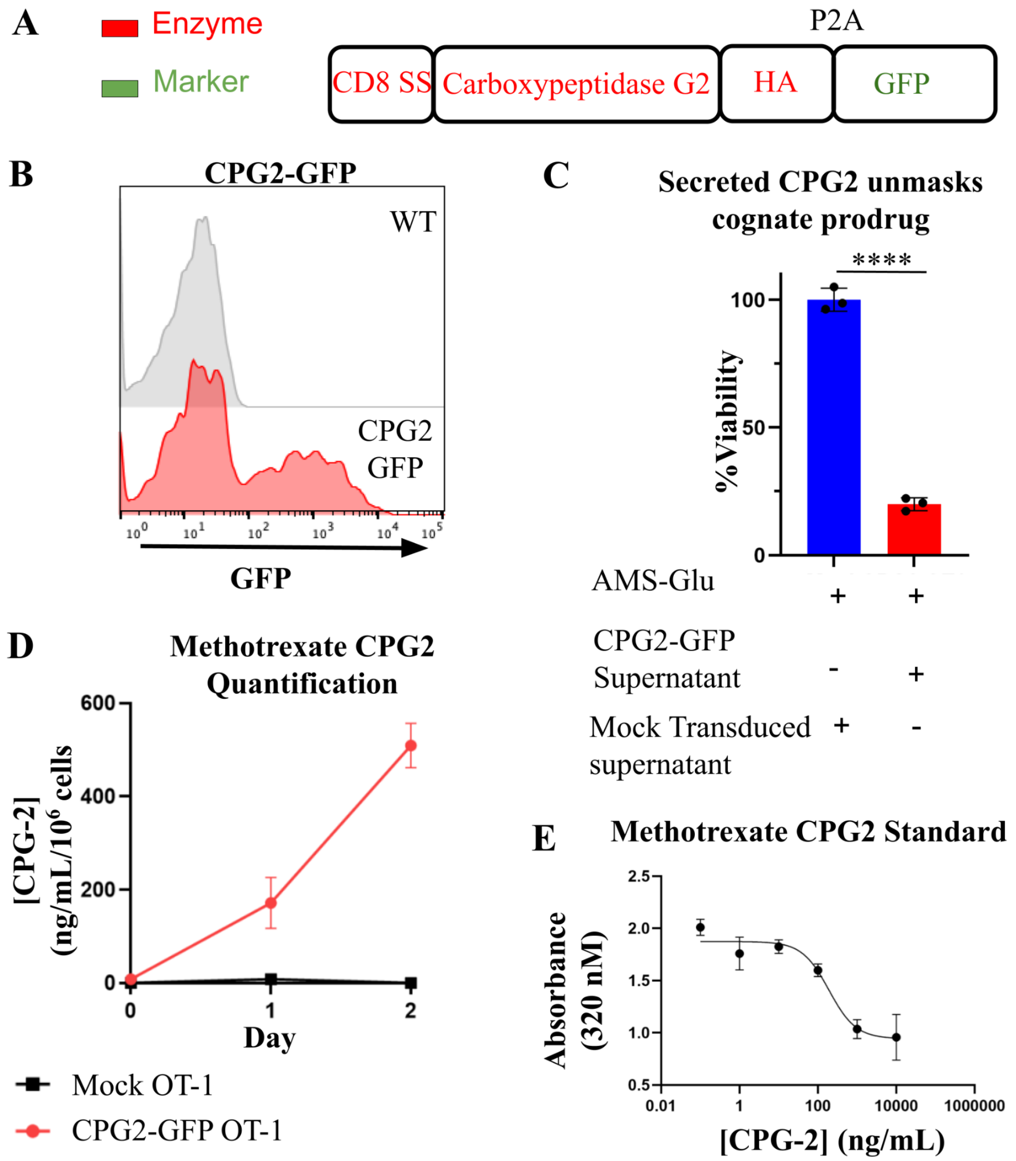

Supplemental Figure 1. OT-1 syngeneic CPG2 SEAKER cells secrete functional enzyme and synergize with prodrug to kill cancer cells. (A) Schematic of SEAKER enzyme secretion cassette for carboxypeptidase G2 (CPG2) with a CD8 signal sequence and a hemagglutinin (HA) tag. (B) Representative flow histogram of murine primary T cell transduction with the CPG2-GFP or β-Lac-GFP plasmid. (C) Supernatant fluid was collected from primary murine T cells transduced with CPG2-GFP or GFP-Luciferase and B16F10 cells were incubated with 100 uM of the cognate prodrug (AMS-GLU), with or without the indicated supernatant fluid. Cell viability was assessed by CellTiter-Glo. (D) Supernatants from mock or CPG2 secreting OT-1 T cells were quantitated for CPG2 using methotrexate and a standard curve. (E) Representative standard curve of recombinant CPG2 with methotrexate.. * = $p<0.05$; ** = $p<0.01$; *** $p< 0.001$; **** = $p<0.0001$

bioRxiv preprint doi: https://doi.org/10.1101/2023.04.05.535717; this version posted May 1, 2023. The copyright holder for this preprint (which was not certified by peer review) is the author/funder, who has granted bioRxiv a license to display the preprint in perpetuity. It is made available under aCC-BY 4.0 International license.

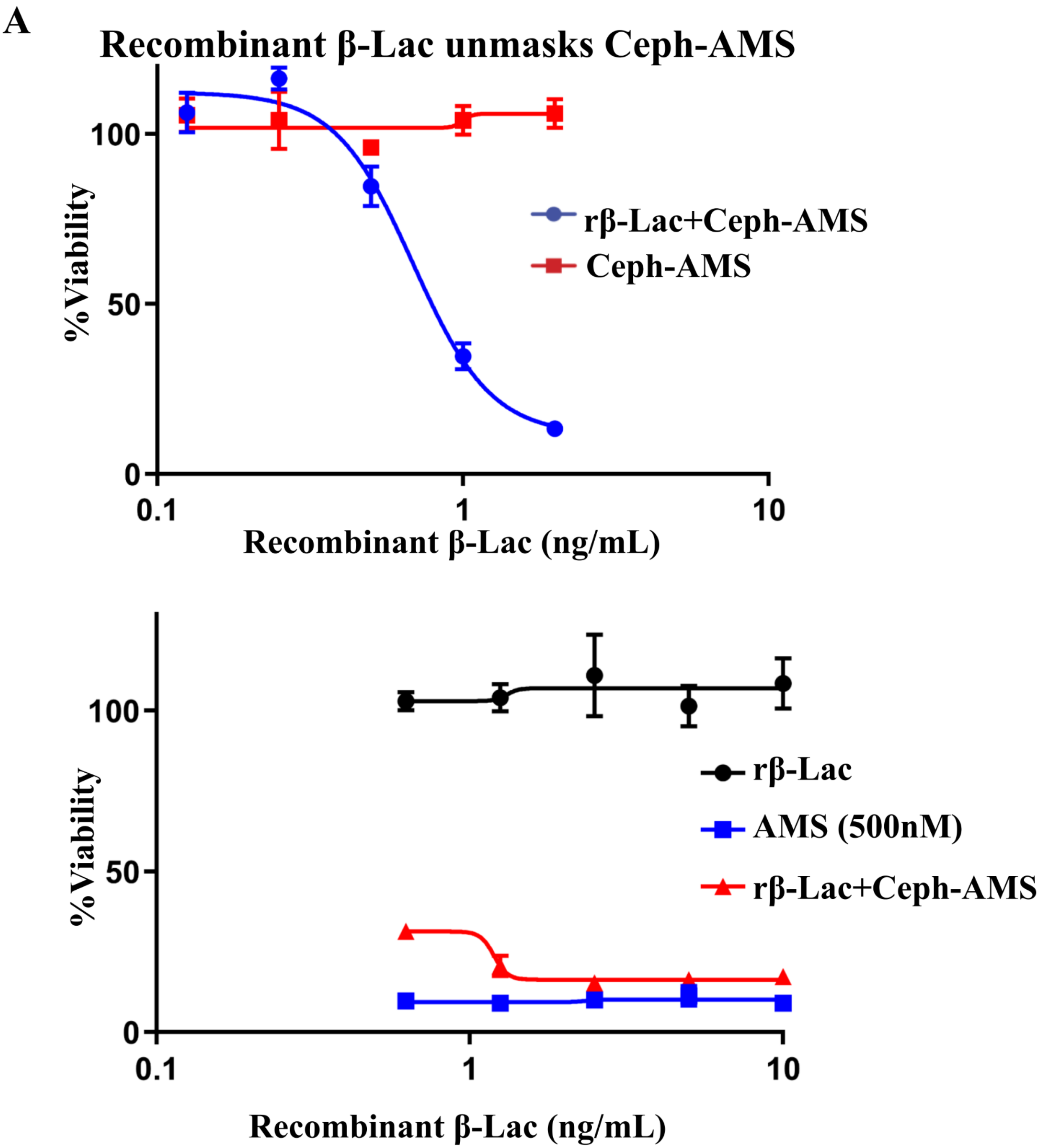

Supplemental Figure 2. Recombinant β-Lactamase unmasks Ceph-AMS to release AMS drug. Set-2 cancer cells were incubated with 500 nM Ceph-AMS and indicated concentrations of recombinant β-Lactamase, and viability of Set-2 cells were assessed by Cell-Titer Glo after 48 hours.

bioRxiv preprint doi: https://doi.org/10.1101/2023.04.05.535717; this version posted May 1, 2023. The copyright holder for this preprint (which was not certified by peer review) is the author/funder, who has granted bioRxiv a license to display the preprint in perpetuity. It is made available under aCC-BY 4.0 International license.

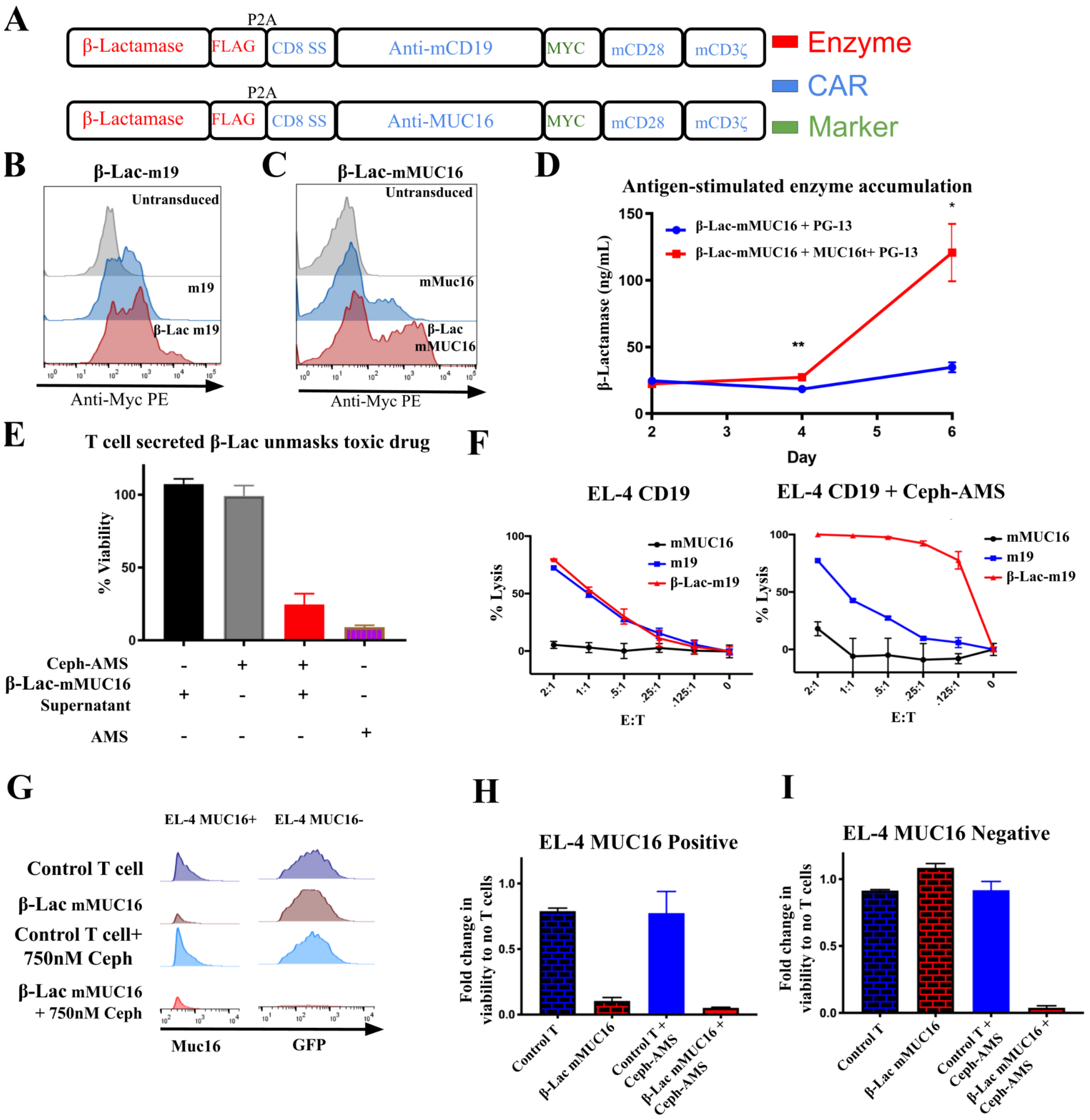

Supplemental Figure 3. Secreted β-Lac can be integrated with various antigen targeting moieties expressed in murine T cells. (A) Secreted β-Lac SEAKER constructs with anti-murine CD19 (top) or anti-MUC16 (bottom) CARs and myc tags. The CAR signaling domains include murine CD28 and murine CD3ζ. (B) β-Lac-m19 and (C) β-Lac-mMUC16 transduction of primary murine T cells. (D) 1 x 10^6 β-Lac-mMUC16 SEAKERs were co-cultured with MUC16 positive or negative PG-13 cells. Enzyme secretion was quantified using the β-Lac substrate nitrocefin compared to a standard curve using recombinant β-Lac. (E) ID8 ovarian cancer cells were incubated as indicated with 500 nM Ceph-AMS prodrug, β-Lac-mMUC16 cell supernatant, or 500 nM AMS parent drug for 48 h and viability was determined by luminescence. (F) β-Lac-m19 or

bioRxiv preprint doi: https://doi.org/10.1101/2023.04.05.535717; this version posted May 1, 2023. The copyright holder for this preprint (which was not certified by peer review) is the author/funder, who has granted bioRxiv a license to display the preprint in perpetuity. It is made available under aCC-BY 4.0 International license.

wild-type m19 cells were incubated at various concentrations with 2 x 10^4 EL-4 CD19+ cells for 24 h, with or without 500 nM Ceph-AMS. Viability was assessed by luminescence compared to untreated cells. (G) β-Lac-mMUC16 cells were co-cultured with a 10% MUC16+ and 90% MUC16-EL-4 cell mixture, with or without 750 nM Ceph-AMS for 24 h. MUC16– and MUC16+ cell populations were assessed by flow cytometry. (H) Quantification of panel G, where total count per MUC16– cell populations were divided by untreated samples to determine percent viability. (I) Quantification of panel G for MUC16+ cell populations. Some of the anti-mMUC16 SEAKER data presented here was also published as a supplemental figure in Gardner et. al. 2022(7).

bioRxiv preprint doi: https://doi.org/10.1101/2023.04.05.535717; this version posted May 1, 2023. The copyright holder for this preprint (which was not certified by peer review) is the author/funder, who has granted bioRxiv a license to display the preprint in perpetuity. It is made available under aCC-BY 4.0 International license.

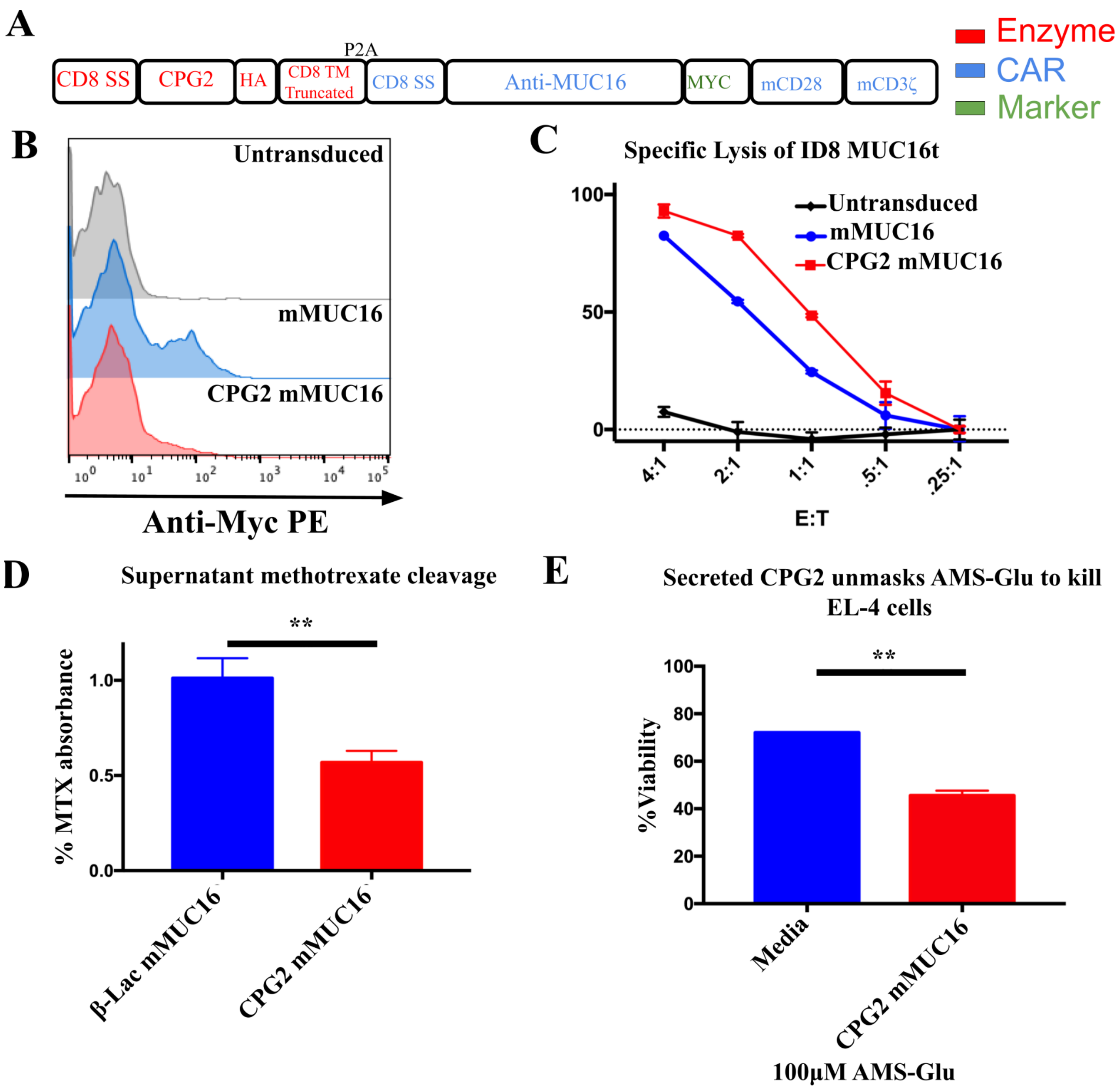

Supplemental Figure 4. Carboxypeptidase G2 secretion by anti-MUC16 CAR-T cells synergizes with Glu-AMS prodrug to kill cancer cells. (A) Secreted CPG2 SEAKER construct with anti-MUC16 CAR. CAR signaling domains include murine CD28 and murine CD3ζ. (B) CPG2-mMUC16 transduction efficiency of primary murine T cells. This CPG2 construct has a lower transduction efficiency and requires more optimization. (C) Cytotoxicity of wild-type anti-mMUC16 and CPG2-mMUC16 SEAKERs compared using a ID8 MUC16+ cell line as target. (D) Supernatant fluid from CPG2 mMUC16 cells was mixed with the CPG2 substrate methotrexate, and loss of methotrexate absorbance was measured. β-Lac-mMUC16 supernatant fluid was used as a negative control that should not cleave methotrexate. (E) Supernatant fluid from CPG2-mMUC16 cells was mixed with 100 µM AMS-Glu and cultured with EL-4 cells for 48 h.

bioRxiv preprint doi: https://doi.org/10.1101/2023.04.05.535717; this version posted May 1, 2023. The copyright holder for this preprint (which was not certified by peer review) is the author/funder, who has granted bioRxiv a license to display the preprint in perpetuity. It is made available under aCC-BY 4.0 International license.

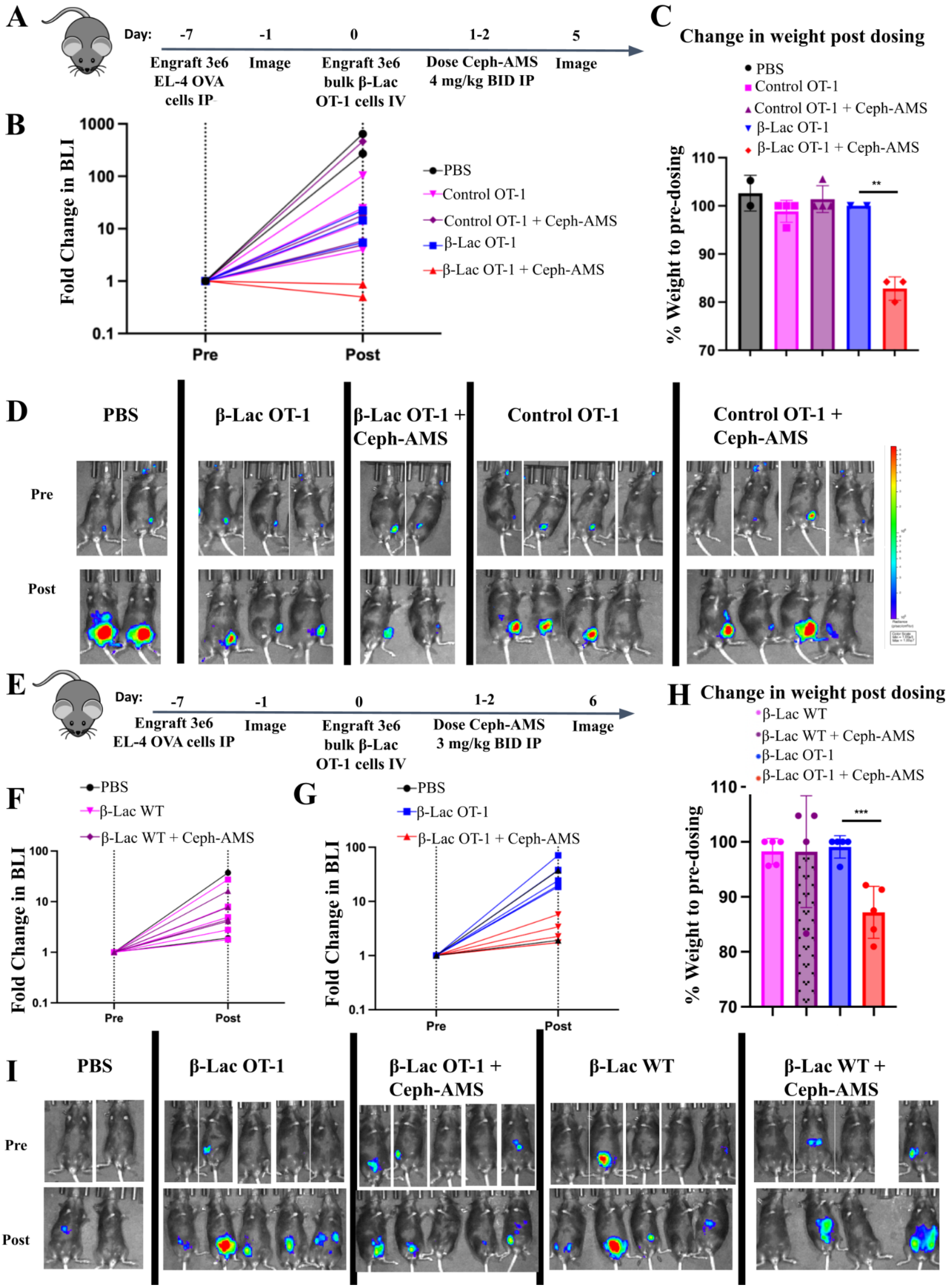

bioRxiv preprint doi: https://doi.org/10.1101/2023.04.05.535717; this version posted May 1, 2023. The copyright holder for this preprint (which was not certified by peer review) is the author/funder, who has granted bioRxiv a license to display the preprint in perpetuity. It is made available under aCC-BY 4.0 International license.

Supplemental Figure 5. Extended data for figure 1E-F. β-Lac OT-1 SEAKER cells unmask Ceph-AMS prodrug in a peritoneal tumor model. (A) Schematics of intraperitoneal proof-of-concept efficacy syngeneic model. EL-4 OVA cells were engrafted *IP* on day -7. BLI imaging was performed one day before T cell engraftment *IP*. Ceph-AMS was given at 4 mg/kg *IP* for three consecutive doses on days 1 and 2 post T cell engraftment. Imaging was performed on day 5. (B) Graph of fold change in tumor BLI pre and post drugging for experiment (A). Two mice in the combination group died from toxicity. (C) Quantification of change in weight pre and post dosing for experiment (A). (D) BLI imaging of pre and post dosing for experiment (A). (E) Replicate experiment at a lower Ceph-AMS dose. Mice were treated as in (A) using 3 mg/kg Ceph-AMS, and imaged on day 6. (F) Quantification of fold change in BLI of the control WT T cells secreting β-lac for experiment (E). (G) Quantification of fold change in BLI of β-lac OT-1 T cells for experiment in (E). (H) Quantification of change in weight pre and post dosing for experiment (E). (I) BLI imaging of pre and post dosing for experiment (E).

bioRxiv preprint doi: https://doi.org/10.1101/2023.04.05.535717; this version posted May 1, 2023. The copyright holder for this preprint (which was not certified by peer review) is the author/funder, who has granted bioRxiv a license to display the preprint in perpetuity. It is made available under aCC-BY 4.0 International license.

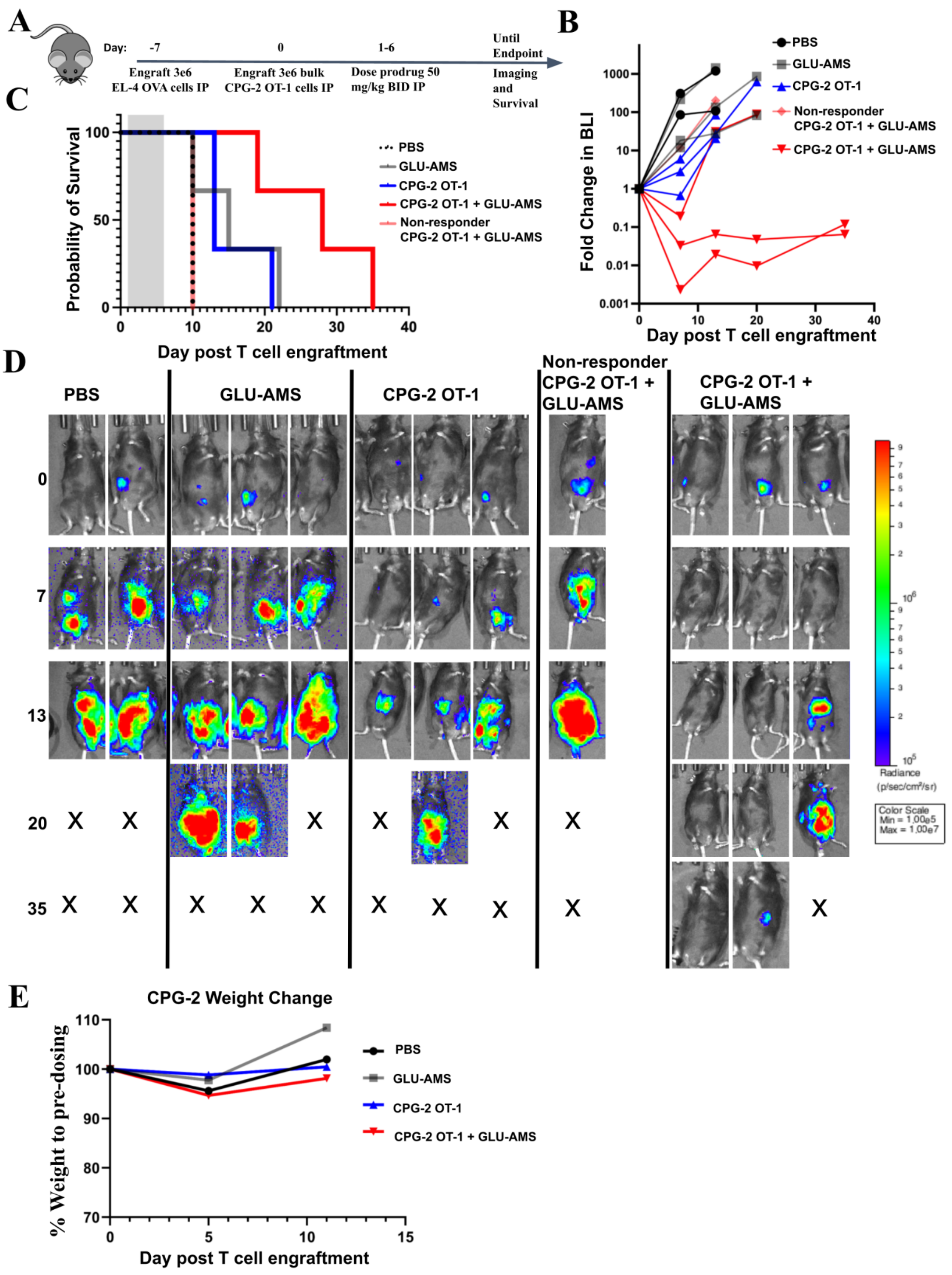

Supplemental Figure 6. CPG2 OT-1 cells unmask AMS-glu prodrug to delay tumor growth *in vivo*. (A) Schematic for testing CPG2 OT-1 SEAKER cells. 3 x 10^6 EL-4 OVA cells were engrafted *IP* at day -7. Mice were imaged and engrafted with bulk 3 x 10^6 OT-1 cells secreting CPG2 on day 0. Mice were given 50 mg/kg *IP*, *BID* from days 1-6. Images were taken of day 7. One mouse that received the CPG combination treatment did not respond to the initial T cell engraftment, and was moved to a non-responder group. This mouse died at the same time as the mice in the PBS treated group. (B) Fold change in BLI for experiment (A). (C) Survival of experiment (A). (D) BLI imaging of experiment (A) An X indicates mouse death. (E) Mean weight values in grams for treatment groups in experiment (A).

bioRxiv preprint doi: https://doi.org/10.1101/2023.04.05.535717; this version posted May 1, 2023. The copyright holder for this preprint (which was not certified by peer review) is the author/funder, who has granted bioRxiv a license to display the preprint in perpetuity. It is made available under aCC-BY 4.0 International license.

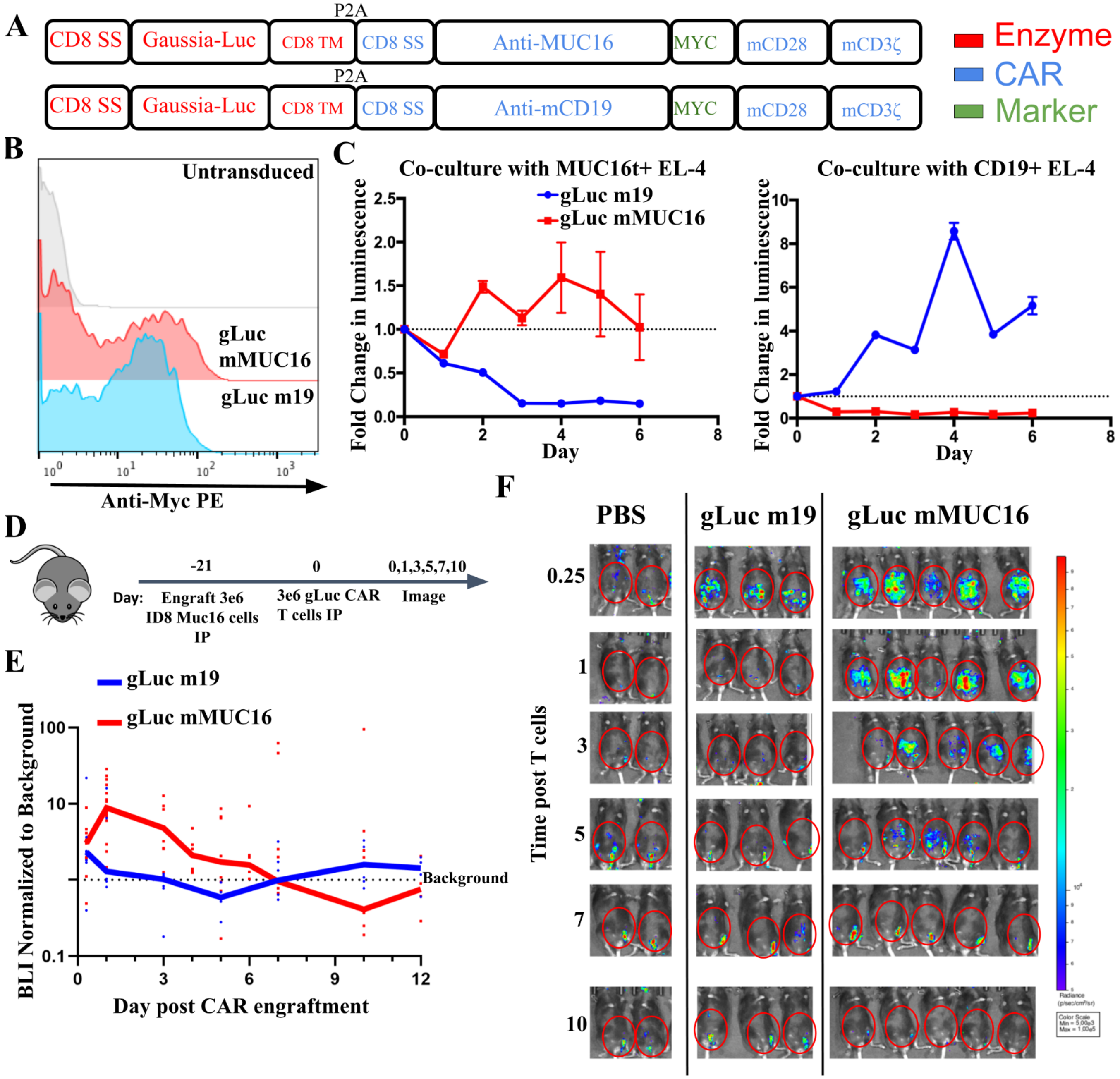

Supplemental Figure 7. Gaussia luciferase enables tracking of murine T cells in a syngeneic mouse model. (A) Schematic of gaussia luciferase (gLuc) constructs alongside the anti-MUC16 CAR (top) or anti-murine CD19 (bottom) with murine CD28 and murine CD3ζ. (B) Transduction of primary murine T cells with gLuc labeled CARs. (C) gLuc CAR were cocultured with EL-4 cells expressing cognate antigen as indicated, in triplicate. (D) Schematic for gLuc tracking in a peritoneal ovarian tumor model. C57BL/6 mice received 3 x 10^6 ID8 cells *IP* at day -21. On day 0, 3 x 10^6 of indicated gLuc CAR T cells were injected *IP* with 100 µg of coelenterazine per mouse, with an exposure of 60 seconds. Images were taken serially at indicated days. (E) Graph of background normalized BLI for the on-target gLuc-mMUC16 (N=17) and off-target gLuc-m19 (N=7) CARs. (F) Representative images of experiment in D.

bioRxiv preprint doi: https://doi.org/10.1101/2023.04.05.535717; this version posted May 1, 2023. The copyright holder for this preprint (which was not certified by peer review) is the author/funder, who has granted bioRxiv a license to display the preprint in perpetuity. It is made available under aCC-BY 4.0 International license.

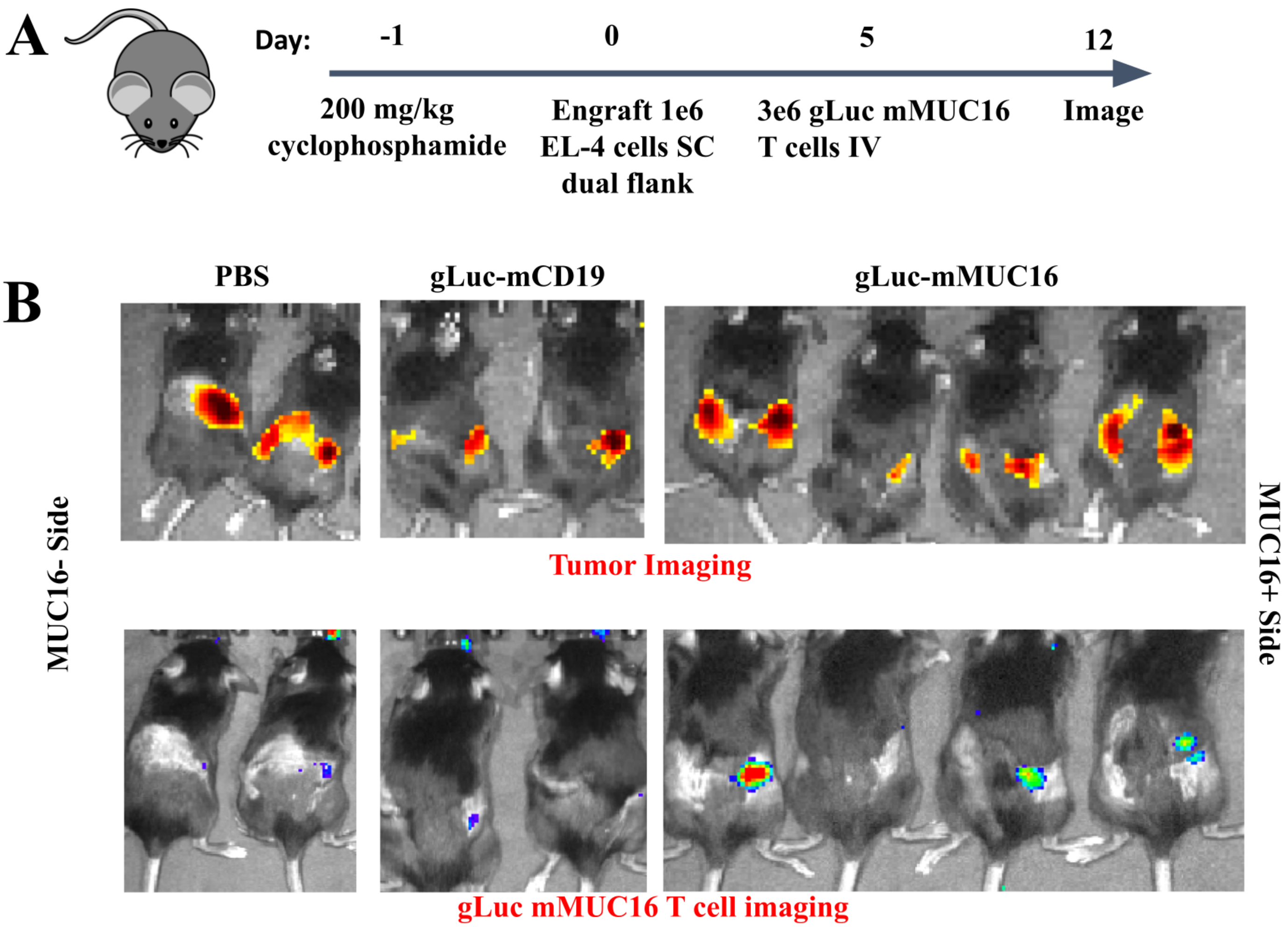

Supplemental Figure 8. Anti-MUC16 CAR T cells home to antigen positive tumors. (A) Schematic where C57BL/6 mice were preconditioned with cyclophosphamide, followed by a subcutaneous engraftment of 1 x 10^6 EL4 MUC16- (left flank of mice) or MUC16+ (right flank of mice) tumors. At day 5, 3 x 10^6 gLuc-mMUC16 cells were engrafted and imaged on day 12. (B) Images of experiment (A). Mice are imaged prone, so the right flank is depicted on the right hand side.

bioRxiv preprint doi: https://doi.org/10.1101/2023.04.05.535717; this version posted May 1, 2023. The copyright holder for this preprint (which was not certified by peer review) is the author/funder, who has granted bioRxiv a license to display the preprint in perpetuity. It is made available under aCC-BY 4.0 International license.

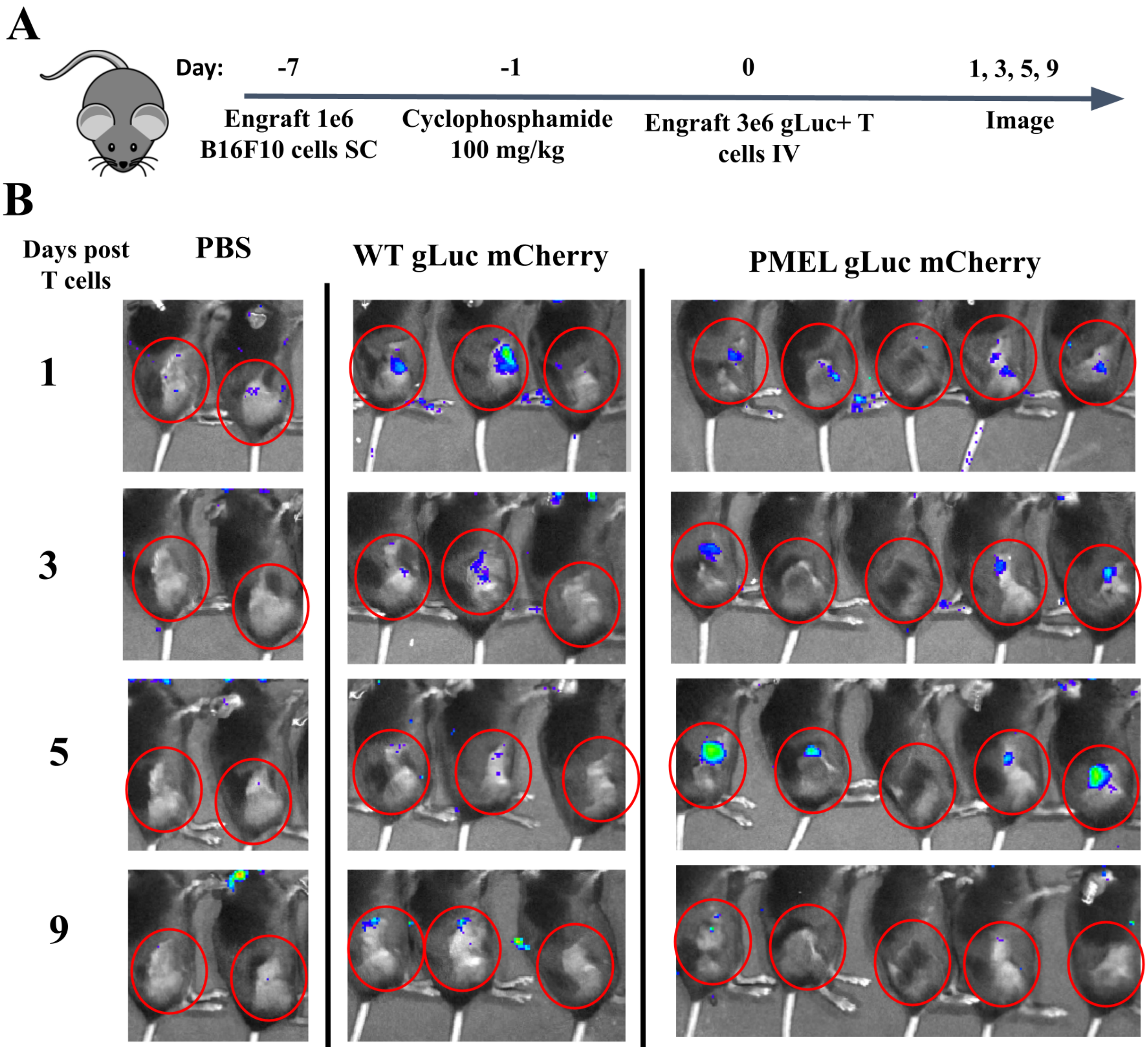

Supplemental Figure 9. PMEL T cells home to gp100 antigen positive B16F10 tumors. (A) Schematic where C57BL/6 mice received *SC* engraftment of 1 x 10^6 B16F10 cells at day -7. Cyclophosphamide was injected *IP* at day -1. PMEL gLuc T cells were then engrafted and T cells were imaged serially through injection of 100ug coelentrazine. (B) Images of experiment (A). Due to retro-orbital injection, non-specific signal may be noted near the eyes.

bioRxiv preprint doi: https://doi.org/10.1101/2023.04.05.535717; this version posted May 1, 2023. The copyright holder for this preprint (which was not certified by peer review) is the author/funder, who has granted bioRxiv a license to display the preprint in perpetuity. It is made available under aCC-BY 4.0 International license.

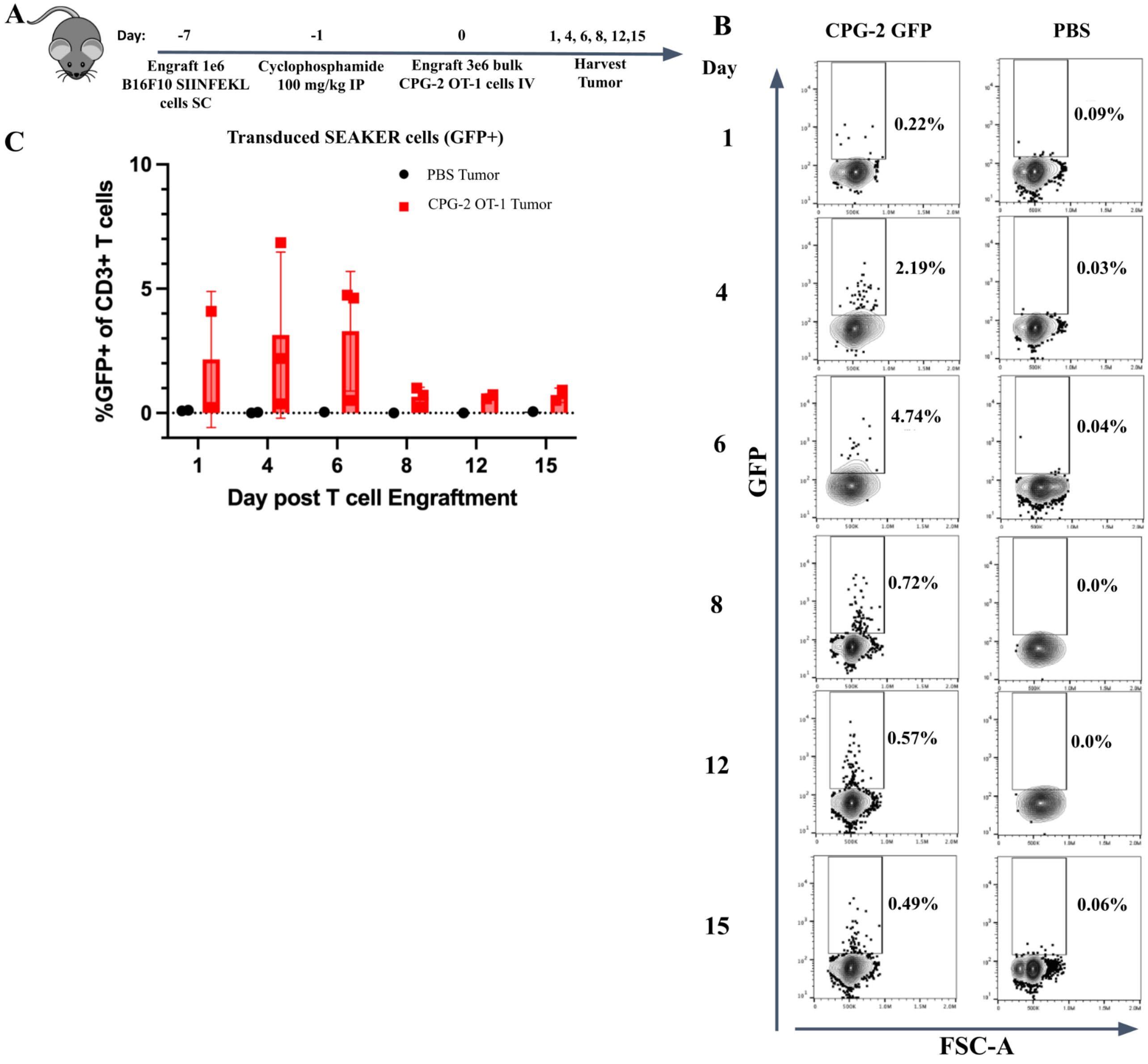

Supplemental Figure 10. CPG2 OT-1 T cells home to antigen positive B16F10 SIINFEKL tumors. (A) Schematic where C57BL/6 mice received *SC* engraftment of 1 x 10^6 B16F10 SIINFEKL cells at day -7. 100 mg/kg cyclophosphamide was injected *IP* at day -1. CPG2 OT-1 T cells were then engrafted and tumors were harvested serially at indicated time point. (B) Representative flow images of GFP+ cells of all CD3+ cells. (C) Graph of (B) across all replicates.

bioRxiv preprint doi: https://doi.org/10.1101/2023.04.05.535717; this version posted May 1, 2023. The copyright holder for this preprint (which was not certified by peer review) is the author/funder, who has granted bioRxiv a license to display the preprint in perpetuity. It is made available under aCC-BY 4.0 International license.

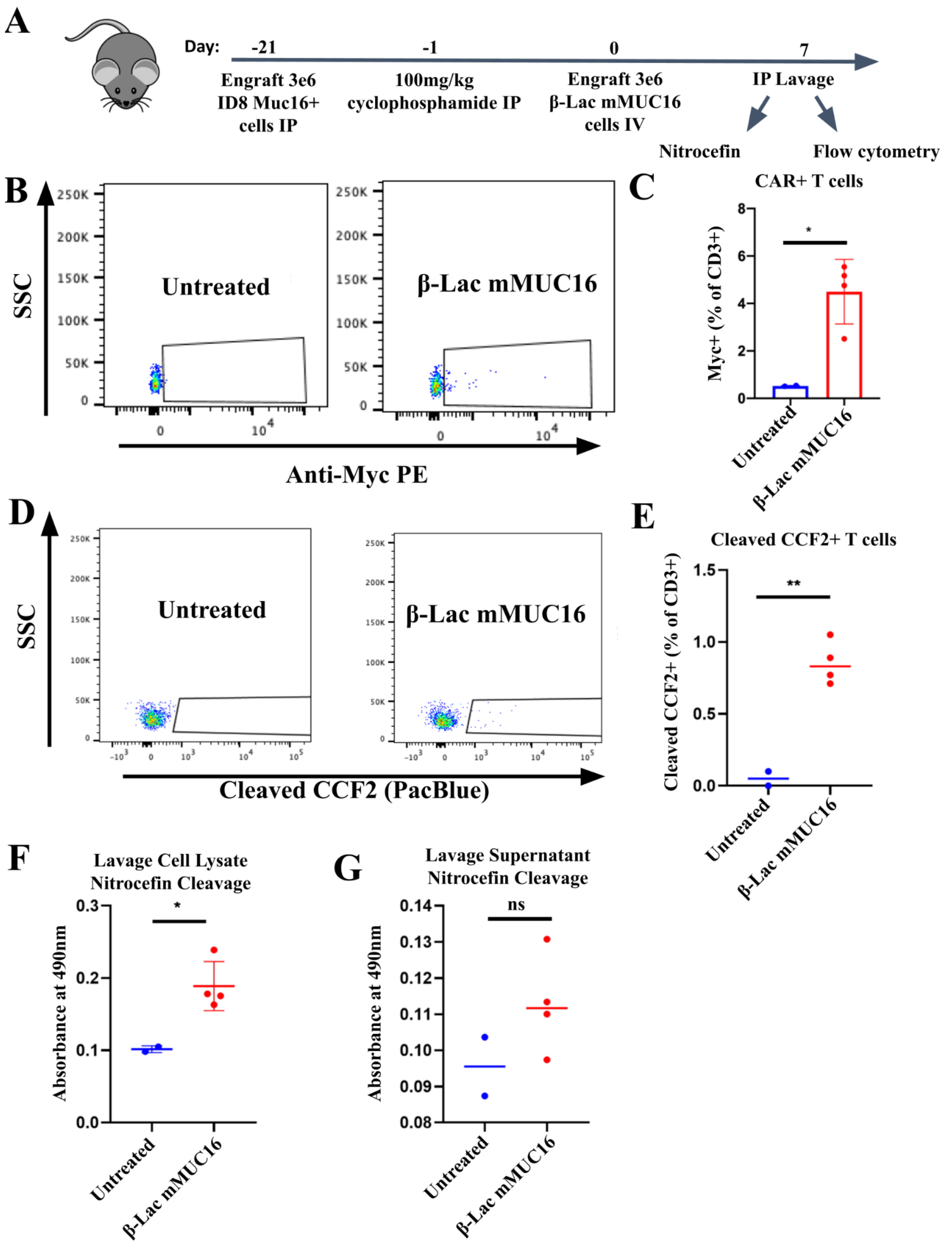

Supplemental Figure 11. β-lac-mMUC16 SEAKER cells deliver β-Lac to peritoneal ovarian tumors. (A) C57BL/6 mice were engrafted with 3 x 10^6 ID8 ovarian tumor cells *IP* at day -21. Mice were preconditioned with 100 mg/kg cyclophosphamide at day -1. On day 0, 3 x 10^6 β-Lac-mMUC16 SEAKERs were engrafted retro-orbitally. Peritoneal lavages were performed on day 7 for flow cytometry and nitrocefin enzyme activity. (B) Flow cytometry of myc staining for CAR+ T cells. (C) Quantification of (B). (D) Flow cytometry of cleaved CCF2 staining, indicating β-Lac activity. (E) Quantification of (D). (F) Cell pellets from peritoneal lavages were lysed and mixed with nitrocefin. A graph of raw absorbance at 490 nm is displayed. (G) Supernatant from the peritoneal lavages were mixed with nitrocefin and raw absorbance at 490 nm was measured.

bioRxiv preprint doi: https://doi.org/10.1101/2023.04.05.535717; this version posted May 1, 2023. The copyright holder for this preprint (which was not certified by peer review) is the author/funder, who has granted bioRxiv a license to display the preprint in perpetuity. It is made available under aCC-BY 4.0 International license.

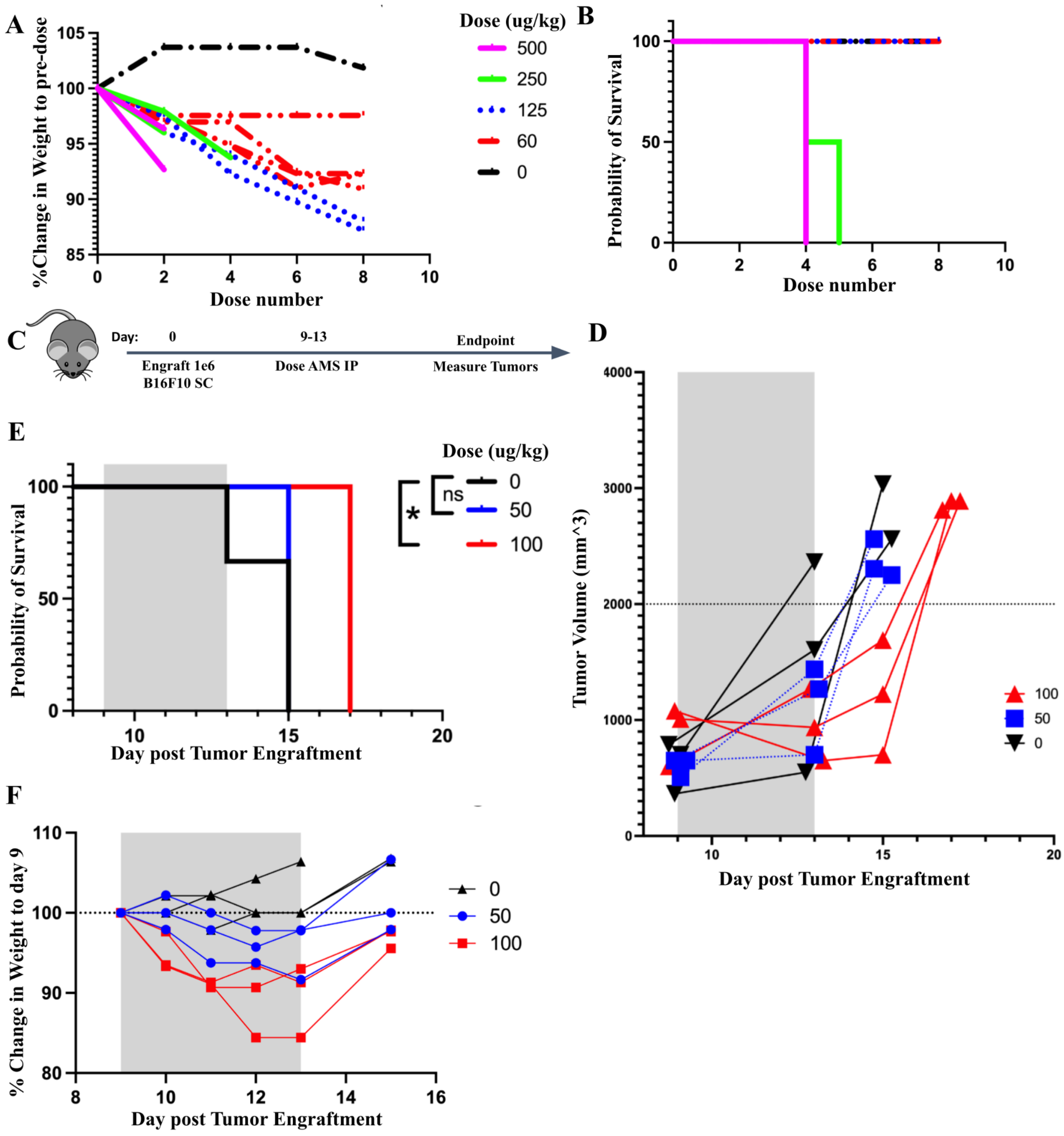

Supplemental Figure 12. AMS is toxic at therapeutic doses. (A) A dose titration of AMS was performed on C57BL/6 mice. A graph of % change in weight is displayed for each dose (µg/kg). (B) Survival of mice treated with indicated concentrations of AMS (µg/kg). (C) Schematic showing mice engrafted with 1 x 10^6 B16F10 cells *SC* followed by *BID* dosing of AMS. (D) Tumor measurements of experiment in (C). (E) Survival of mice from experiment in (C). (F) Percent change in weight for mice from experiment in (C).

bioRxiv preprint doi: https://doi.org/10.1101/2023.04.05.535717; this version posted May 1, 2023. The copyright holder for this preprint (which was not certified by peer review) is the author/funder, who has granted bioRxiv a license to display the preprint in perpetuity. It is made available under aCC-BY 4.0 International license.

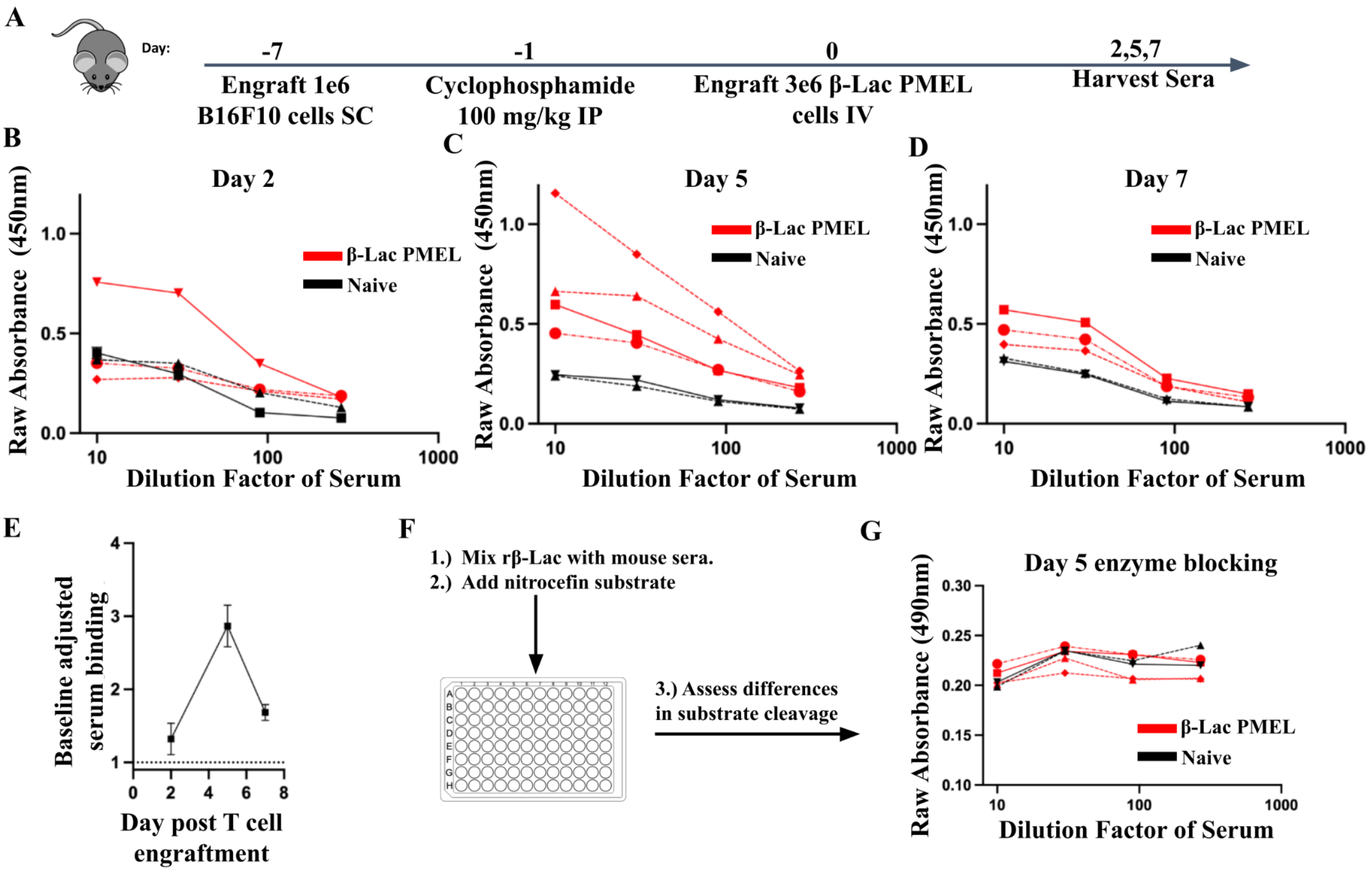

Supplemental Figure 13. β-Lac SEAKER cells elicit a humoral immune response in C57BL/6 mice. (A) Mice were engrafted with 1 x 10^6 B16F10 cells *SC* at day -7, 100 mg/kg of cyclophosphamide on day -1, and β-Lac PMEL T cells on day 0. Sera were collected at indicated time points. (B-D) Serum samples were tested for reactivity to recombinant untagged β-Lac at day 2 (B), day 5 (C), and day 7 (D). Raw absorbance is depicted at 450 nm. (E) Average serum binding was normalized to naive mice at the 1:30 serum dilution. (F) Schematic depicting β-Lac enzyme functional assay in which recombinant β-Lac was mixed with serum samples and nitrocefin was added to assess change in activity. (G) Graph depicting raw absorbance values for recombinant β-Lac incubated with indicated serum samples and concentrations.

bioRxiv preprint doi: https://doi.org/10.1101/2023.04.05.535717; this version posted May 1, 2023. The copyright holder for this preprint (which was not certified by peer review) is the author/funder, who has granted bioRxiv a license to display the preprint in perpetuity. It is made available under aCC-BY 4.0 International license.

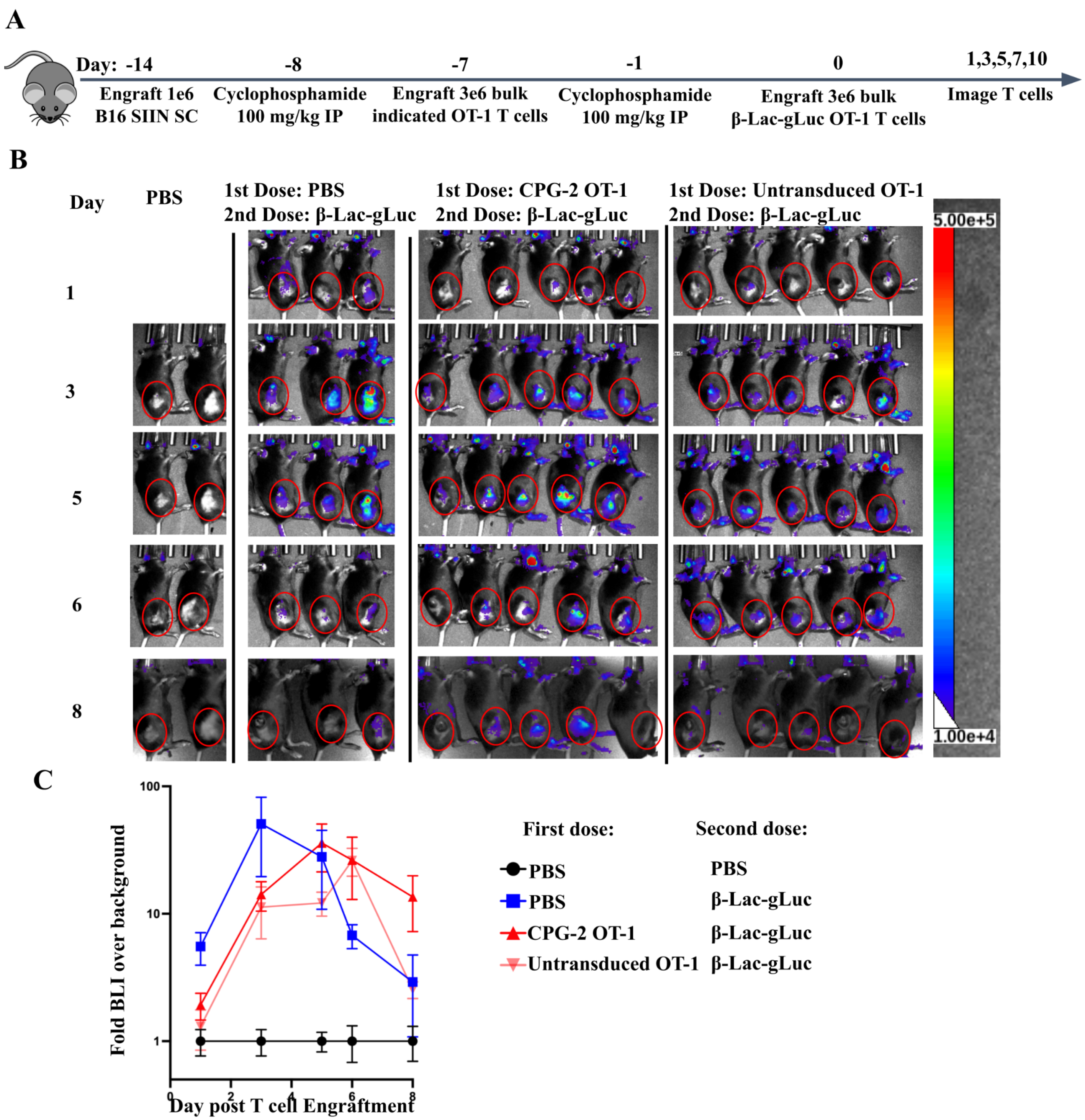

Supplemental Figure 14. β-Lac SEAKER cells can be re-engrafted with CPG2 SEAKER cells. (A) C57BL/6 mice were engrafted with 1 x 10^6 B16F10 SIINFEKL cells *SC* at day -14, pretreated with 100 mg/kg cyclophosphamide at day -8 and treated with 3 x 10^6 indicated OT-1 cells retro-orbitally on day -7. Mice were retreated with 100 mg/kg cyclophosphamide on day -1 and re-engrafted with trackable gLuc+ β-Lac SEAKER cells on day 0. Mice were imaged serially. (B) T cell bioluminescent imaging from experiment (A). (C) Quantification of background adjusted BLI values from (B).
